# Supplementary material for: Mycobacterial biotin synthases require an auxiliary protein to convert dethiobiotin into biotin
Source: Nat Commun. 2024 May 16;15:4161. doi: 10.1038/s41467-024-48448-1 (PMC11099021; doi:10.1038/s41467-024-48448-1)
Supplement: Supplementary file 1 — Supplementary Information [file 41467_2024_48448_MOESM1_ESM.pdf]

Supplementary Figures

Mycobacterial biotin synthases require an auxiliary protein to convert dethiobiotin into biotin

Di Qu[1], Peng Ge[2], Laure Botella[1], Sae Woong Park[1], Ha-Na Lee[1], Natalie Thornton[1], James M. Bean[3], Inna V. Krieger[4], James C. Sacchettini[4], Sabine Ehrt[1], Courtney Aldrich[2], Dirk Schnappinger[1]

1 Department of Microbiology and Immunology, Weill Cornell Medicine, New York, NY 10021, USA.

2 Department of Medicinal Chemistry, University of Minnesota, Minneapolis, MN 55455, USA

3 Sloan Kettering Institute, Memorial Sloan Kettering Cancer Center, 1275 York Avenue, New York, NY 10021, USA

4 Texas A&M University, Department of Biochemistry and Biophysics, ILSB 2138, 301 Old Main Dr, College Station, Texas 77843-3474, USA

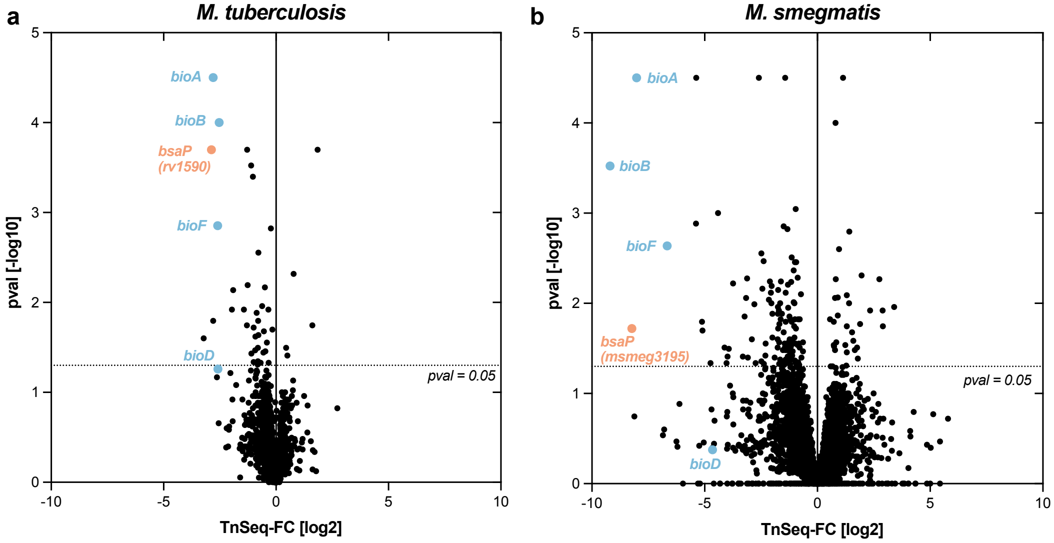

Supplementary Fig. 1. Identification of mycobacterial genes required for growth in the absence of extrabacterial biotin. Volcano plots showing transposon insertions that change growth of *M. tuberculosis* (**a**) or *M. smegmatis* (**b**) in biotin free meida. Negative fold changes indicate mutations that cause attenuation in the absence of biotin. Blue dots identify genes known to be required to synthesize biotin in *M. tuberculosis* and *M. smegmatis.* The only other gene that was required by both species to grow normally in biotin free media is shown in orange.

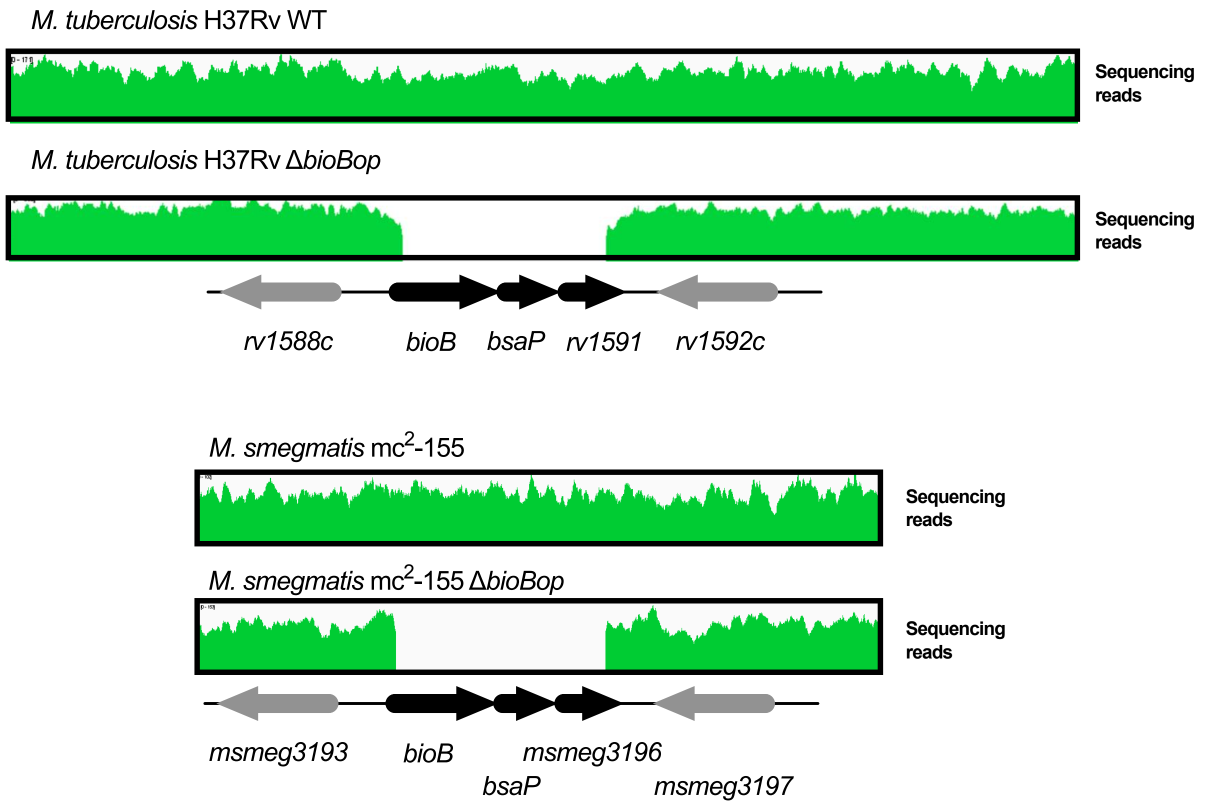

Supplementary Fig. 2. Verification of Δ*bioBop* in *M. tuberculosis* and *M. smegmatis* by whole-genome sequencing. Mutants were verified by the lack of sequencing reads mapping to the gene deletions.

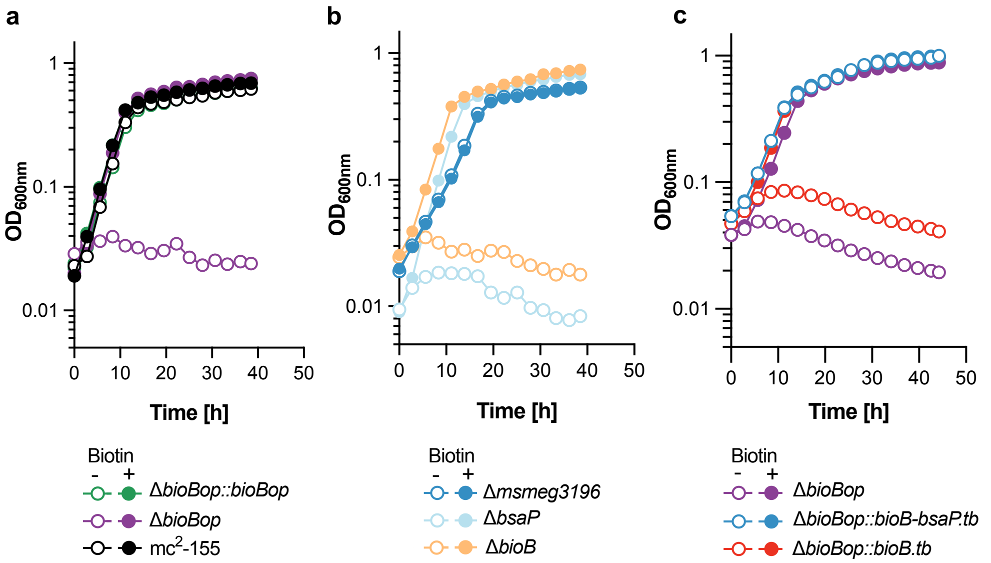

Supplementary Fig. 3. ***BsaP* is required by *M. smegmatis* to grow without extrabacterial biotin. a-c**, Growth in modified 7H9 medium with (filled symbols) and without biotin (open symbols) was assessed by optical density measurements. The Δ*bioBop* strain shown in **a** and **c** represents *M. smegmatis* Δ*bioBop*. The other strains were constructed by complementing *M. smegmatis* Δ*bioBop* with the required genes from *M. smegmatis* (**b**) or *M. tuberculosis* (**c**). Source data are provided as a Source Data file.

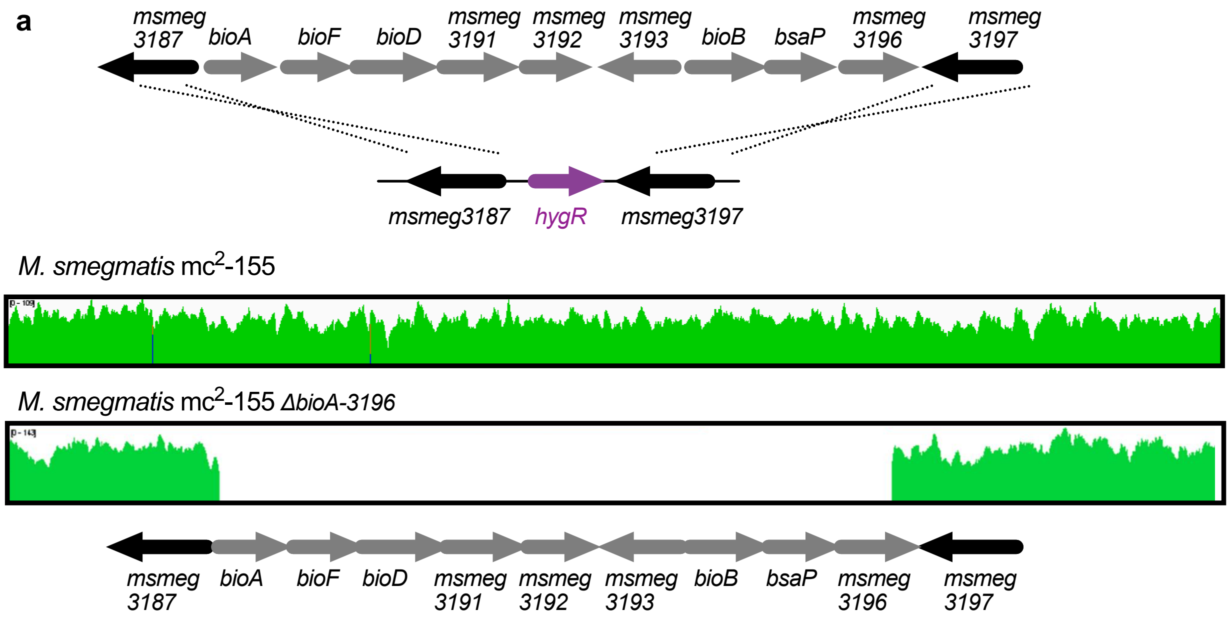

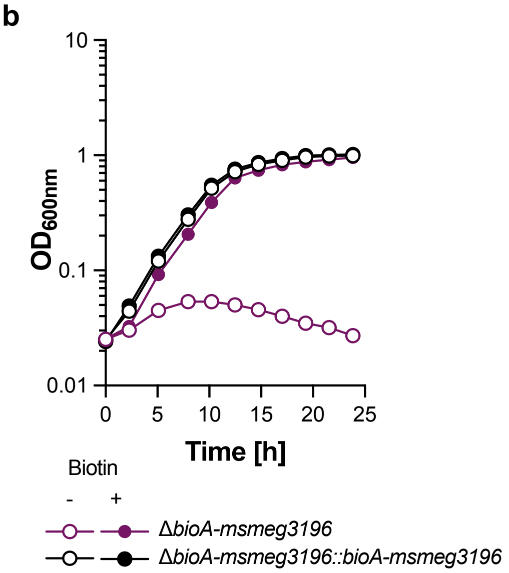

Supplementary Fig. 4. Construction and verification of *M. smegmatis* mutant *ΔbioA-msmeg3196*. a, Design and verification by whole-genome sequencing of *ΔbioA-msmeg3196.* Green line represent sequencing reads. **b,** Growth in modified 7H9 medium with (filled symbols) and without biotin (open symbols). Source data are provided as a Source Data file (**b**).

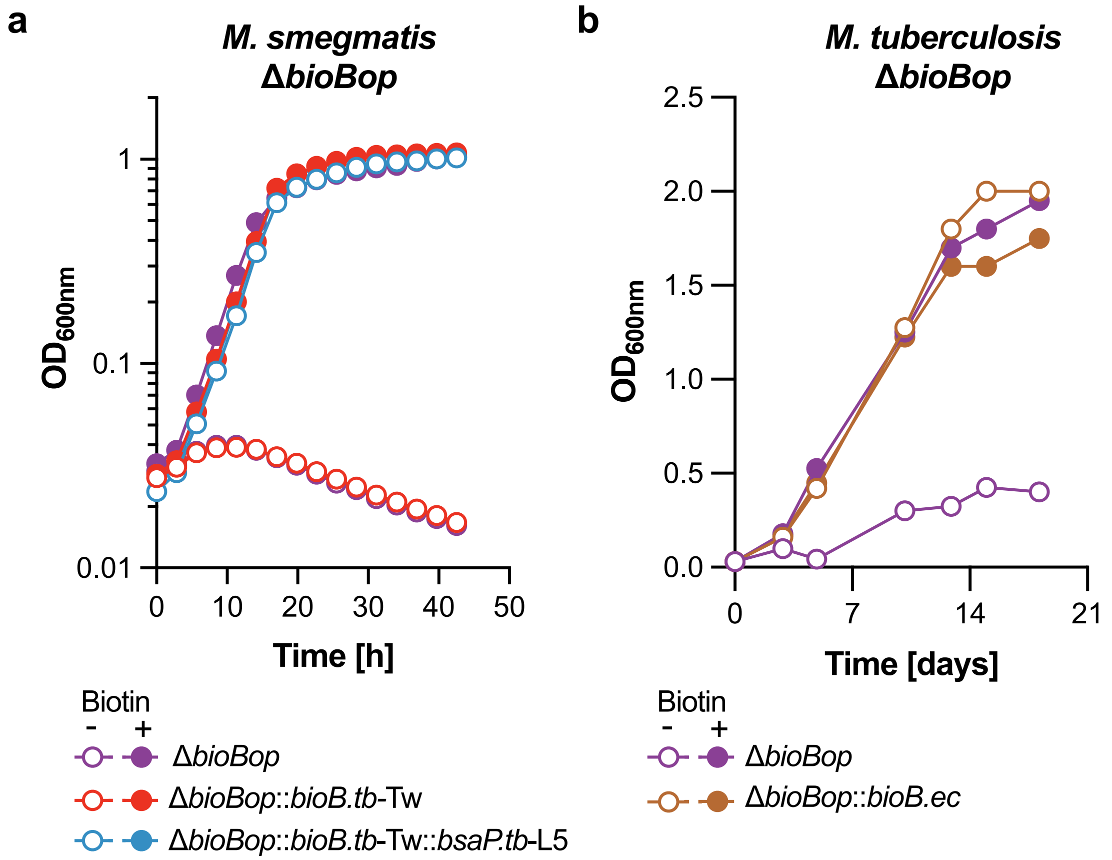

Supplementary Fig. 5. BsaPtb complements *M. smegmatis ΔbioPop* in trans and is dispensable for complementation of *M. tuberculosis ΔbioPop* by *bioB.ec*. a, **b**, Growth in modified 7H9 medium with (filled symbols) and without biotin (open symbols) was assessed by optical density measurements. Source data are provided as a Source Data file.

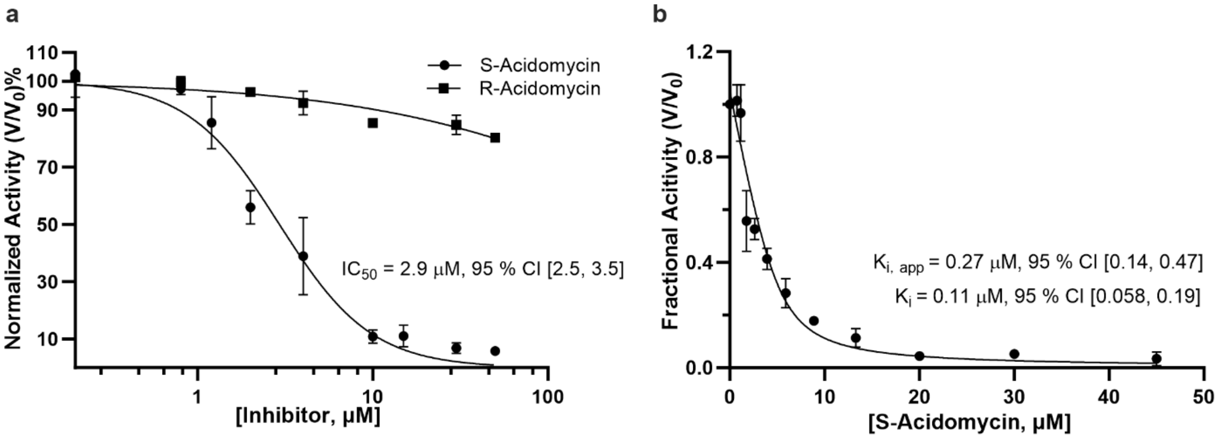

Supplementary Fig. 6. Inhibition of BioB-BsaP by acidomycin. a. The IC_{50} of *S*-acidomycin was determined using 5 μM BioB(dimer)-BsaP and fixed saturating concentration of DTB (10 μM) and SAM (100 μM), The observation that the IC_{50} of *S*-acidomycin is approximately one-half enzyme concentration indicates tight-binding inhibition, thus requiring analysis using the Morrison equation. **b.**The apparent K_i ($K_{i,app}$) of S-acidomycin was determined using otherwise identical conditions, but using 1.5-fold concentration dilution series of *S*-acidomycin. The data was fitted to the Morrison quadratic equation in Prism. Acidomycin exhibits competitive inhibition with respect to DTB for *Ec*BioB,[29] thus the $K_{i,app}$ for *Mt*BioB can be corrected to provide the K_i shown in panel b, using the Cheng-Prusoff equation, $K_i = \frac{K_{i,app}}{(1+[DTB]/K_m}$. **a,b**,n=3. Data are presented as mean values +/- SD.Source data are provided as a Source Data file.

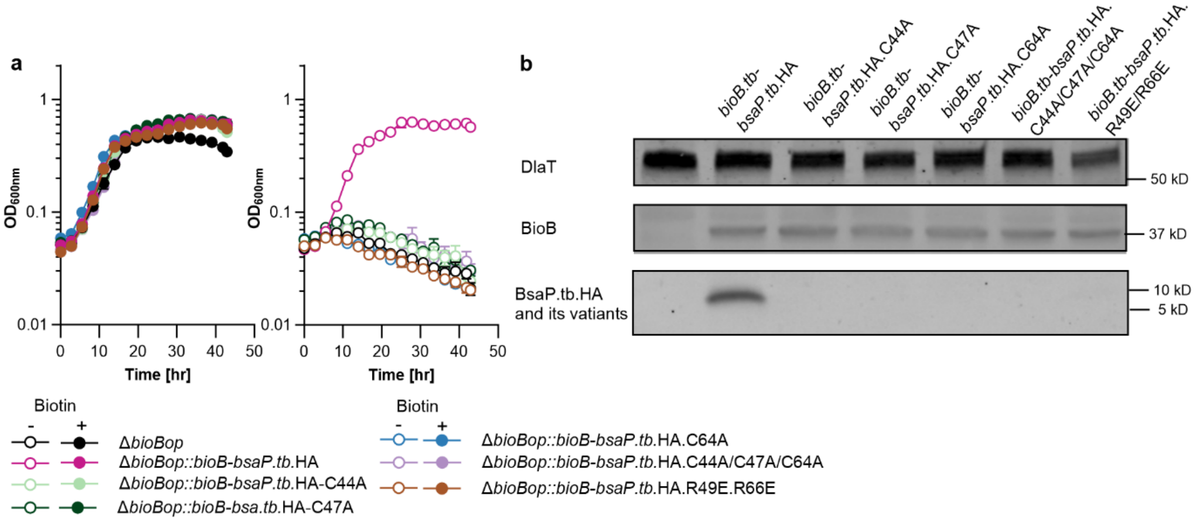

Supplementary Fig. 7. Analysis of BsaP.tb variants in *M. smegmatis*. a. Growth of BsaP.tb variants in *M.smegmatis.* **b**. Immunoblots for expression of BioB.tb, BsaP.tb, and BsaP.tb variants in *M.smegmatis.* C-terminal hemagglutinin (HA) tags were added to all BsaP.tb variants to enable their detection with HA antibodies. DlaT was used as the loading control in this experiment. The entire blot for this experiment is shown in Supplementary Fig.11d. Results represent at least 3 independent experiments (**b**). Source data are provided as a Source Data file (**a**).

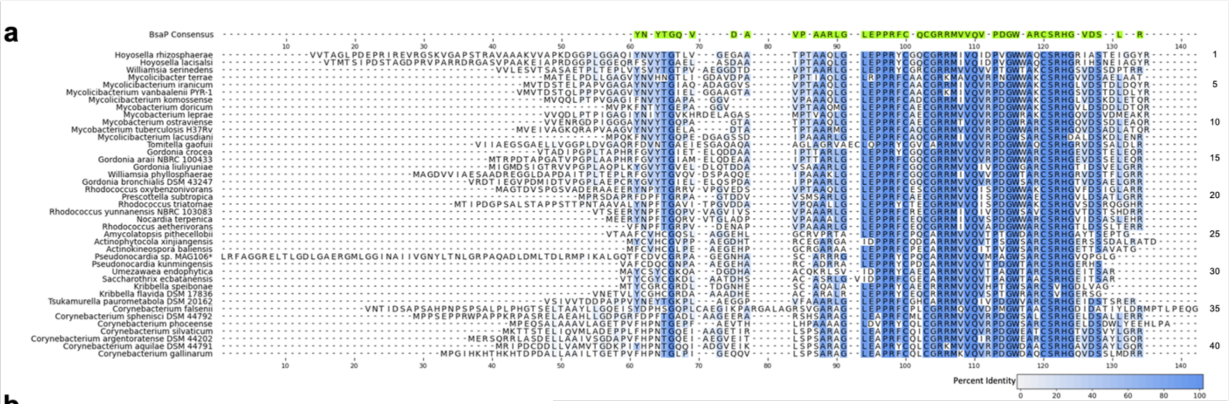

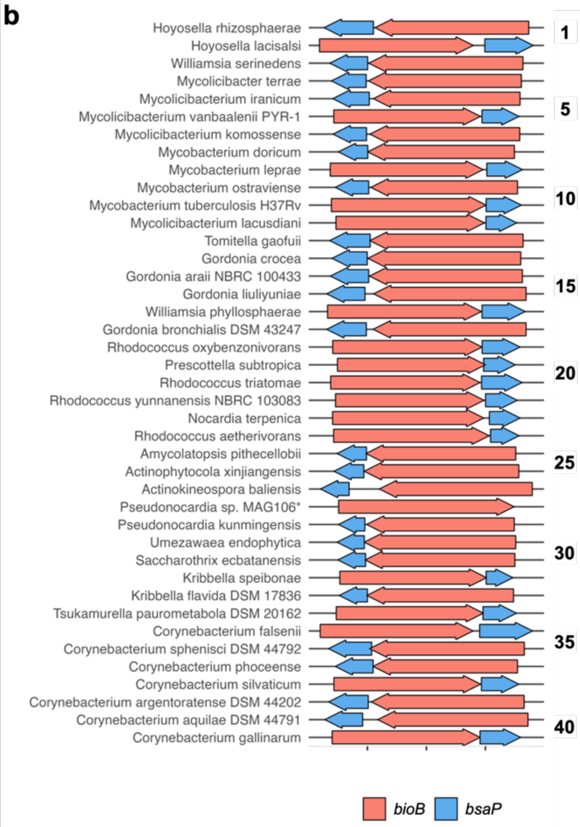

Supplementary Fig. 8. BsaP homologs. a. BsaP protein homolog multiple sequence alignment of the proteins shown in Fig. 5d. Residues are highlighted by adherence to consensus sequence, and consensus sequence is shown at the top of the alignment. **b**. BioB and BsaP homolog annotation and relative positioning showing that *bsaP* genes are almost always positioned downstream of *bioB*.

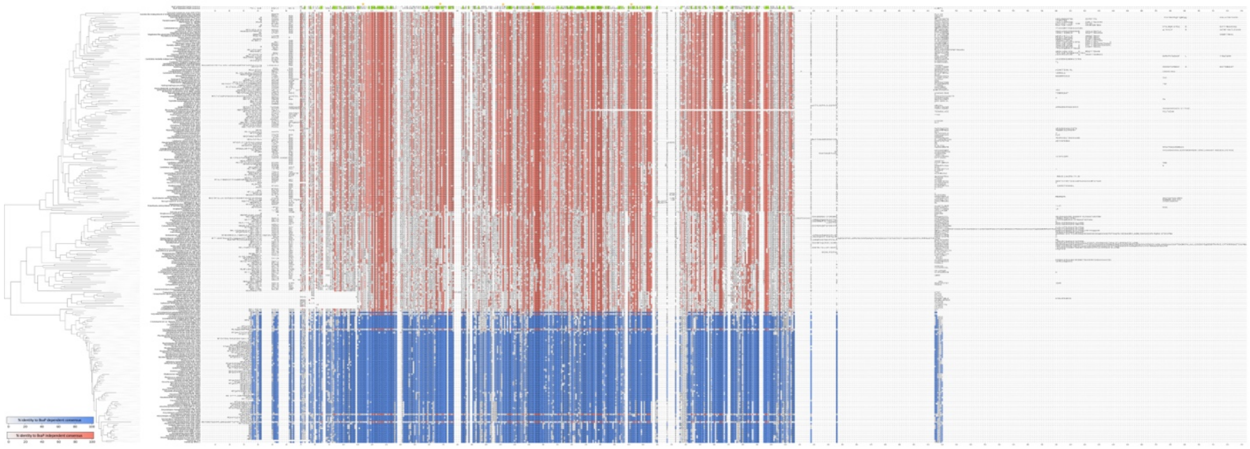

Supplementary Fig. 9. **Phylogenetic tree and sequence alignment of biotin synthases.** Biotin synthases encoded by genes located within 1kb of genes encoding BsaP homologs are shown in blue. Sequences of biotin synthases encoded in genomes that do not encode a BsaP homolog downstream of *bioB* are shown in red. Residues discussed in the main text are indicated by yellow dots above the consensus sequences shown on top of the alignment.

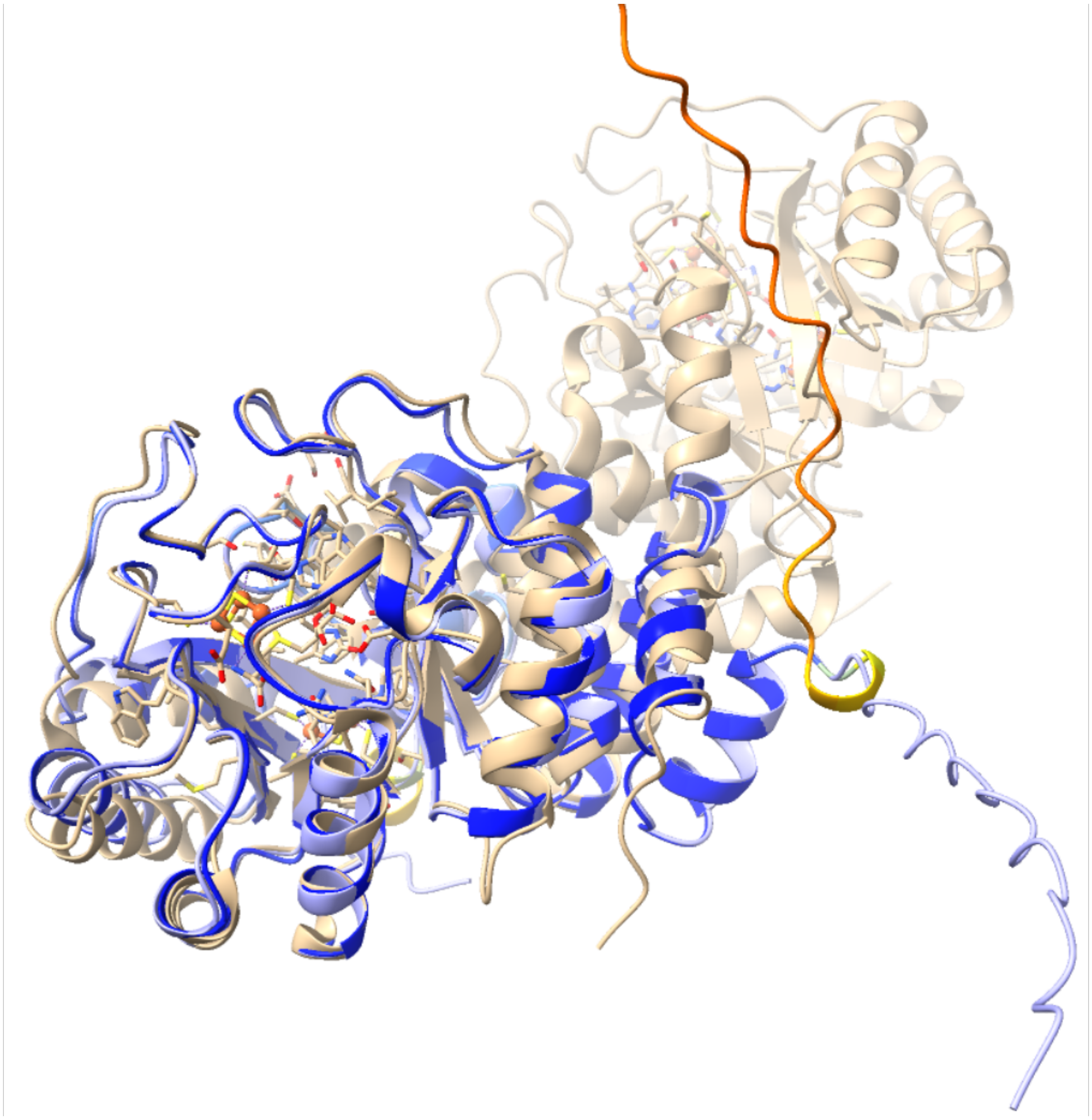

Supplementary Fig. 10. **Comparison of BioB.ec structure (1R30, tan) with AF predictions for BioB.tb (lilac ribbon) and BioB.sm (colored by AF prediction confidence score).**

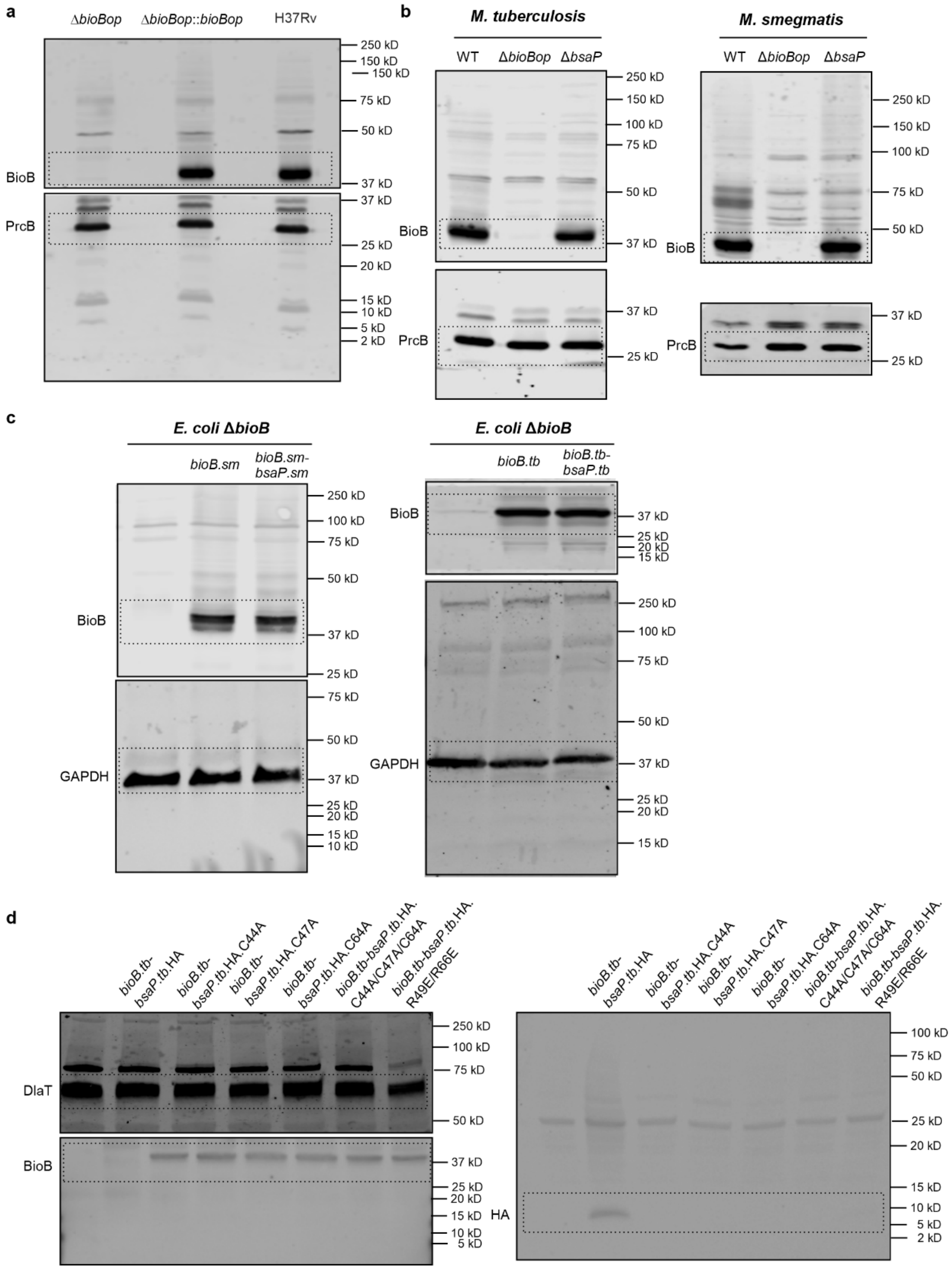

Supplementary Fig. 11. Full western blots images. a. Immunoblots for BioB and PrcB (loading control) in *M. tuberculosis*, refers to **Fig. 1c** . **b.** Immunoblots for expression of BioB and PrcB (loading control) in *M. smegmatis,* refers to **Fig. 3a**. **c.** Immunoblots for expression of BioB and GAPDH (loading control) in *E. coli*, refers to Fig. 3b. **d.** Immunoblots for expression of BioB.tb, BsaP.tbHA, BsaP.tb variants, and DlaT (loading control) *in M.smegmatis,* refers to **Extended Data Fig. 5a**. Results represent at least 3 independent experiments.
