# Supplementary material for: Mycobacterial biotin synthases require an auxiliary protein to convert dethiobiotin into biotin
Source: Nat Commun. 2024 May 16;15:4161. doi: 10.1038/s41467-024-48448-1 (PMC11099021; doi:10.1038/s41467-024-48448-1)
Supplement: Supplementary file 3 — Description of Additional Supplementary Files [file 41467_2024_48448_MOESM3_ESM.pdf]

Description of Additional Supplementary Files:

Supplementary Dataset 1: Media.

Supplementary Dataset 2: Plasmids.

Supplementary Dataset 3: Strains.
