# Supplementary material for: Mycobacterial biotin synthases require an auxiliary protein to convert dethiobiotin into biotin
Source: Nat Commun. 2024 May 16;15:4161. doi: 10.1038/s41467-024-48448-1 (PMC11099021; doi:10.1038/s41467-024-48448-1)
Supplement: Supplementary file 5 — Reporting Summary [file 41467_2024_48448_MOESM5_ESM.pdf]

nature portfolio

Corresponding author(s): Dirk Schnappinger

Last updated by author(s): April 8, 2024

Reporting Summary

Nature Portfolio wishes to improve the reproducibility of the work that we publish. This form provides structure for consistency and transparency in reporting. For further information on Nature Portfolio policies, see our Editorial Policies and the Editorial Policy Checklist.

Statistics

For all statistical analyses, confirm that the following items are present in the figure legend, table legend, main text, or Methods section.

n/a	Confirmed	
[ ]	[x]	The exact sample size (*n*) for each experimental group/condition, given as a discrete number and unit of measurement
[ ]	[x]	A statement on whether measurements were taken from distinct samples or whether the same sample was measured repeatedly
[ ]	[x]	The statistical test(s) used AND whether they are one- or two-sided<br>*Only common tests should be described solely by name; describe more complex techniques in the Methods section.*
[x]	[ ]	A description of all covariates tested
[ ]	[x]	A description of any assumptions or corrections, such as tests of normality and adjustment for multiple comparisons
[ ]	[x]	A full description of the statistical parameters including central tendency (e.g. means) or other basic estimates (e.g. regression coefficient) AND variation (e.g. standard deviation) or associated estimates of uncertainty (e.g. confidence intervals)
[ ]	[x]	For null hypothesis testing, the test statistic (e.g. *F*, *t*, *r*) with confidence intervals, effect sizes, degrees of freedom and *P* value noted<br>*Give P values as exact values whenever suitable.*
[x]	[ ]	For Bayesian analysis, information on the choice of priors and Markov chain Monte Carlo settings
[x]	[ ]	For hierarchical and complex designs, identification of the appropriate level for tests and full reporting of outcomes
[x]	[ ]	Estimates of effect sizes (e.g. Cohen's *d*, Pearson's *r*), indicating how they were calculated

Our web collection on statistics for biologists contains articles on many of the points above.

Software and code

Policy information about availability of computer code

Data collection	Prism (version 9.3.1, GraphPad); BV-BRC
Data analysis	Prism (version 9.3.1, GraphPad); MUSCL; IQTree; TRANIST Version 3.2.3 TnSeq analysis software tool which includes the pre-processing tool TPP, custom code archived in Zenodo with the identifier https://doi.org/10.5281/zenodo.10912991

For manuscripts utilizing custom algorithms or software that are central to the research but not yet described in published literature, software must be made available to editors and reviewers. We strongly encourage code deposition in a community repository (e.g. GitHub). See the Nature Portfolio guidelines for submitting code & software for further information.

Data

Policy information about availability of data

All manuscripts must include a data availability statement. This statement should provide the following information, where applicable:

- Accession codes, unique identifiers, or web links for publicly available datasets
- A description of any restrictions on data availability
- For clinical datasets or third party data, please ensure that the statement adheres to our policy

Data generated during the study are available within the paper and/or its supplement. Source data are provided with this paper. M. tuberculosis H37Rv or M. smegmatis mc2-155 reference genomes (NC_018143.1, NC_008596.1, respectively) were used to map transposon-chromosome-junctions.

Research involving human participants, their data, or biological material

Policy information about studies with human participants or human data. See also policy information about sex, gender (identity/presentation), and sexual orientation and race, ethnicity and racism.

Reporting on sex and gender	Not applicable
Reporting on race, ethnicity, or other socially relevant groupings	Not applicable
Population characteristics	Not applicable
Recruitment	Not applicable
Ethics oversight	Not applicable

Note that full information on the approval of the study protocol must also be provided in the manuscript.

Field-specific reporting

Please select the one below that is the best fit for your research. If you are not sure, read the appropriate sections before making your selection.

☒ Life sciences ☐ Behavioural & social sciences ☐ Ecological, evolutionary & environmental sciences

For a reference copy of the document with all sections, see nature.com/documents/nr-reporting-summary-flat.pdf

Life sciences study design

All studies must disclose on these points even when the disclosure is negative.

Sample size	No calculations were performed a priori to determine sample size. Sample sizes followed common standards in the field and are specified.
Data exclusions	No data were excluded.
Replication	Experiments were performed at least twice. All replication attempts were successful.
Randomization	Samples were alocated into experimental groups randomly.
Blinding	No blinding was performed by the investigators in this study. Blinding was not necessary because the performed experiments were not subject to group bias.

Reporting for specific materials, systems and methods

We require information from authors about some types of materials, experimental systems and methods used in many studies. Here, indicate whether each material, system or method listed is relevant to your study. If you are not sure if a list item applies to your research, read the appropriate section before selecting a response.

Materials & experimental systems

n/a	Involved in the study	
☐	☒	Antibodies
☒	☐	Eukaryotic cell lines
☒	☐	Palaeontology and archaeology
☒	☐	Animals and other organisms
☒	☐	Clinical data
☒	☐	Dual use research of concern
☒	☐	Plants

Methods

n/a	Involved in the study	
☒	☐	ChIP-seq
☒	☐	Flow cytometry
☒	☐	MRI-based neuroimaging

Antibodies

Antibodies used	Rabbit Anti-BioB.tb, was made by GenScript (Order ID: U8595BJ180-1; Lot No.: A317030069)<br>Rabbit Anti-BioB.sm, was made by GenScript (Order ID: U7648CC310-7; Lot No.: A317070075)<br>Mouse Anti-GAPDH, Abcam, Catalog No.: ab125247, Clone name: GA1R, Lot No.: GR3412422-1<br>Mouse Anti-HA, ThermoFisher, Catalog No: 26183, Clone name: 2-2.2.14, Lot No.: TJ272546

Validation	Specificity of the antibody used to detect BioB was validated by (i) binding to recombinant BioB, (ii) detection of a band of the expected molecular weight in extracts from WT M. tuberculosis/ M. smegmatis, and (iii) lack of a BioB band in extracts from bioB deletion mutants of M. tuberculosis and M. smegmatis. Data was shown in Manuscript Fig. 1c (M. tuberculosis BioB) and in Fig. 3a(M. smegmatis BioB). Anti-PrcB and Anti-DlaT antibodies have been previously validated, Anti-PrcB (DOI: https://doi.org/10.1038/s41467-022-29832-1); Anti-DlaT (DOI: https://doi.org/10.1038/ncomms14731). The validation information of Mouse Anti-GAPDH and Mouse Anti-HA were confirmed from manufacture's websites (Mouse Anti-GAPDH: https://www.abcam.com/products/primary-antibodies/gapdh-antibody-ga1r-loading-control-ab125247.html; Mouse Anti-HA: https://www.thermofisher.com/antibody/product/HA-Tag-Antibody-clone-2-2-2-14-Monoclonal/26183).

Plants

Seed stocks	Not applicable
Novel plant genotypes	Not applicable
Authentication	Not applicable
